# Objective and subjective psychosocial outcomes in adults with autism spectrum disorder: A 6-year longitudinal study

**DOI:** 10.1177/13623613211027673

**Published:** 2021-06-25

**Authors:** Anke M Scheeren, J Marieke Buil, Patricia Howlin, Meike Bartels, Sander Begeer

**Affiliations:** 1 VU University Amsterdam, The Netherlands; 2 King’s College London, UK

**Keywords:** autism, adulthood, adult outcomes, employment, independent living, well-being, longitudinal study

## Abstract

Research has shown lower rates of employment and independent living in adults with autism. Many of these findings are based on cross-sectional studies, predominantly involving male participants. In a 6-year longitudinal study, we examined determinants of psychosocial outcomes in 917 adults with autism spectrum disorder (425 men, 492 women, mean age 43.5 years). Most were diagnosed in adulthood and had (above) average intellectual abilities. Via a yearly online survey, participants’ objective psychosocial functioning (based on employment, independent living and friendship) and subjective well-being were assessed. Averaged across the five waves of the study, 86% of the sample showed a fair to very good level of objective psychosocial functioning. Objective psychosocial functioning and subjective well-being were positively correlated, and both improved over time. Lower intellectual ability, more autism traits, co-occurring psychiatric conditions and younger age predicted poorer objective outcomes. More autism traits and co-occurring psychiatric conditions predicted lower subjective well-being. There were no gender differences in initial levels of, or changes in, objective and subjective outcomes over time. This study has identified important risk/protective factors for psychosocial outcomes across early and middle adulthood. In general, the findings offer a more positive outlook for adults with autism and average to high intellectual abilities.

**Lay abstract:**

Previous research has shown that relatively few adults with autism have a paid job or live on their own. However, outcomes also vary a lot and may depend on many different factors. In this study, we examined the level of functioning and happiness of 917 adults with autism (425 men and 492 women) aged 18–65 years. Most of them were of average to high intellectual ability. Over 6 years, we measured whether they had a paid job, close friendships and lived on their own (i.e. their objective functioning). We also measured how happy they felt. Objectively, most autistic adults did fairly to very well. Those with better objective outcomes (e.g. those with paid work) also tended to be happier. Most adults improved in objective functioning and happiness over 6 years. Participants with a lower intellectual ability, more autism traits, mental health problems and younger age had poorer objective outcomes. Participants with more autism traits and mental health problems were less happy. Autistic men and women functioned at similar levels and were equally happy. We found important factors that predict a better (or worse) outcome for autistic adults. Overall, compared with some previous research, our findings give a more positive picture of the outcomes for autistic adults with average to high intellectual abilities.

Many autistic adults^1^ find it difficult to attain socially ‘normative’ life goals such as holding a job, living independently or finding a romantic partner (Billstedt et al., 2011; Bishop-Fitzpatrick et al., 2016; Howlin & Moss, 2012; Roux et al., 2013). Variance in adult outcomes is large, however, with some individuals reaching high levels of independence (Gotham et al., 2015; Helles et al., 2017), whereas others, including those of average or above-average intellectual ability, may require care and support throughout adulthood (Hofvander et al., 2009; Howlin & Moss, 2012; Lord et al., 2020). A recent meta-analysis of 18 studies, involving 1199 autistic adults of mixed intellectual abilities, concluded that around 50% of participants had a (very) poor overall outcome (Mason et al., 2020). An exception to these generally poor outcomes was reported by Gotham et al. (2015). They found that 40% of late-diagnosed, self-reporting adults (n = 255; 64% females) had a regular paid job and just over half lived alone or with a partner. Yet, to date, most studies of adult outcomes are based on cross-sectional data in primarily male samples (Lord et al., 2020) and sample size is typically small. The aim of the present longitudinal study is to explore determinants of psychosocial outcomes in autistic men and women over time.

Psychosocial functioning is a broad term encompassing several domains including, but not limited to, functioning at work, relationship quality, independent living and physical health (Wilson et al., 2015). The most consistent predictors of a positive psychosocial outcome in adulthood include higher intellectual and verbal ability (Howlin et al., 2013; Pickles et al., 2020), fewer autism traits (Deserno et al., 2018; Zimmerman et al., 2018) and no co-occurring psychiatric conditions (Helles et al., 2017; Kraper et al., 2017; Lin & Huang, 2019). Sex differences in psychosocial outcomes are unclear, likely due to the low number of females in most cohorts (Magiati et al., 2014). Employment rates generally appear similar for men and women (Sung et al., 2015; Wei et al., 2015), although Taylor and colleagues (2015) reported higher rates for men. Friendship quality and social skills tend to be higher in women (Head et al., 2014; Hull et al., 2019; Lai et al., 2017). The relationship with age remains uncertain, although there are some indications of improvement over time. Several studies report a reduction in autism severity (e.g. (Howlin et al., 2013; Seltzer et al., 2004) and mental health problems (Lever & Geurts, 2016; Uljarević et al., 2020) from late adolescence/early adulthood onwards. Howlin et al. (2013), in one of the few longitudinal studies involving adults in middle age, found that for most participants, although not all, overall psychosocial outcomes remained stable over time.

Successful psychosocial outcomes in adulthood are typically operationalized in terms of work, housing and marital status. However, as advocates from the autism community emphasize, success may be better defined by subjective well-being or happiness (AUTISTICA, 2016; Lord et al., 2020). Indeed, Deserno et al. (2018) found ‘happiness’ to be a central concept with strong associations with other outcomes among autistic adults. Systematic reviews and a meta-analysis reported a lower subjective quality of life (QoL) in adults with autism compared to the general population (Ayres et al., 2018; van Heijst & Geurts, 2015). This may be linked to their poorer objective outcomes since, in the general population, poorer subjective well-being is highly correlated with lower levels of normative success (Walsh et al., 2018). However, in autism, although some studies reported a positive correlation between objective and subjective outcomes (Lawson et al., 2020; Mason et al., 2018; Moss et al., 2017), others do not (Helles et al., 2017; Pickles et al., 2020). For example, having a regular job or living independently may come at a high personal cost, including increased stress, anxiety or sensory overload (Baldwin & Costley, 2016; Bishop-Fitzpatrick et al., 2016), and thus may negatively affect subjective well-being. Further research is required on the predictors of and associations between objective and subjective psychosocial outcomes in autism.

In the current longitudinal study, we examined levels, change and predictors of objective psychosocial functioning and subjective well-being in a large group of adults (18–65 years) with autism spectrum disorder (ASD). We hypothesized (1) overall improvements in objective and subjective functioning over time; (2) positive associations between objective and subjective functioning; (3) that higher intellectual ability predicts a higher level of and greater improvement in objective psychosocial functioning over time and (4) that the absence of co-occurring psychiatric conditions predicts higher subjective well-being. In addition, we explored the predictive value of age, autism traits, gender, age of ASD diagnosis and parental education in predicting levels of and change in objective and subjective outcomes. Finally, given the broad age range of the sample, we explored whether the predictive value of some variables varied as a function of age (i.e. whether age served as a moderator between predictors and outcomes).

## Method

### Participants

Participants were recruited through the Netherlands Autism Register (NAR) at the Vrije Universiteit Amsterdam. The NAR sends out annual online surveys to autistic adults, parents and legal representatives of individuals with ASD. The survey programme began in 2013 (Wave 1) continuing up to 2018 (Wave 5). Individuals were included in the present study if they: (1) reported a formal ASD diagnosis established by a qualified clinician (e.g. psychiatrist) in a professional setting (e.g. mental healthcare clinic); (2) participated in at least two of the five study waves in the period 2013–2018 and (3) were aged 18–65 years during at least two waves. The final sample consisted of 917 adults (425 men and 492 women) with a mean age of 43.5 years (SD = 12.4) at their most recent wave. Non-binary participants were not included in the analysis because, at the start of the NAR data collection, it was not possible for participants to indicate a gender other than male or female. (Since 2016, the NAR offers the possibility to register as ‘other’ gender. Of the autistic adults who fill in the annual survey, 1% identifies as ‘other’ gender.) Informants at each wave comprised self-reporting adults with ASD (91%–93%) and legal representatives (parents) of an adult with ASD (9%–7%). The large majority of participants (97%) identified as Dutch. Based on available data from 850 adults, the highest finished educational level was low (e.g. pre-vocational secondary school) for 16%; middle (e.g. secondary vocational education) for 38% and high (e.g. higher professional education and university education) for 46% of participants.

Of the 917 participants, a quarter (25.6%) partook in all five waves of the study. Another 17.4% participated in four out of five waves, and 28.0% participated in three out of five waves. Thus, 71.1% had participated in at least three out of five waves of the study. Comparisons of participants with complete data throughout all five waves versus participants who had missing data at any time-point indicated that participants with complete data had higher levels of subjective well-being at the start of the study compared to participants with missing data (complete data: M = 3.1, SD = 1.1; missing data: M = 2.8, SD = 1.1; *t* = 2.55, *p* = 0.011, Cohen’s *d* = 0.233). No differences in mean levels/proportions of objective psychosocial functioning, or any of the predictors (age, gender distribution, autism traits, intellectual ability, age of ASD diagnosis, maternal and paternal educational level and presence of co-occurring psychiatric conditions), between participants with and without missing data were found (all *p*s ⩾ 0.05).

### Instruments

#### Outcome measures

##### Objective psychosocial functioning

Following Howlin et al. (2004), a composite measure of objective psychosocial functioning was based on employment, independent living and friendship ratings; the total score derived from these variables ranged from 0 (very poor outcome) to 8 (very good outcome); see also Table 1.

**Table 1.** Coding of Employment, Independent Living, Friendships and Overall Objective Psychosocial Functioning.

Scale	Coding
Employment scale (0-3)	
Regular paid employment/self-employed for at least 16 hrs. per week; Or studying for at least 24 hours per week	3
Regular paid employment/self-employed for less than 16 hrs. per week; Or non-regular/non-paid employment	2
Not any kind of (paid or unpaid) employment	1
No structural day time activities for 40 hours or more per week	0
Independent living scale (0-3)	
Independently (either alone or with partner and/or children)	3
Independently with some housing assistance	2
With parents/family	1
Form of housing with guidance and/or care; Or a healthcare institution	0
Friendship scale (0-2)	
Close friendships	2
Social contacts (other than parents, siblings or own children), but no close friendships	1
Hardly any social contacts (except for contact with parents, siblings or own children)	0
Overall objective psychosocial functioning (0-8)	
Very good outcome: regular paid job/self-employed for at least 16 hrs. per week, living independently, close friendships	8
Good outcome: one life domain with suboptimal outcome (e.g., social contacts, but no close friendships)	7
Fair outcome: one or more life domains with suboptimal outcome	4-6
Poor outcome: two or all life domains with a relatively poor outcome (e.g., not any kind of (paid or unpaid) employment)	1-3
Very poor outcome: no structural daytime activities, form of housing with guidance or a healthcare institution, hardly any social contacts	0

##### Subjective well-being

Subjective well-being was measured with a closed-ended question: ‘Which of the following statements about your well-being fits you [the person with autism] best?’ Informants selected one of the following statements: ‘Always or almost always happy’ (5), ‘More happy than unhappy’ (4), ‘Equally happy and unhappy’ (3), ‘More unhappy than happy’ (2) and ‘Always or almost always unhappy’ (1). Higher scores indicate higher subjective well-being (Bartels & Boomsma, 2009; Begeer et al., 2017).

##### Predictor variables

Predictor variables include age (in years), gender (0 = female; 1 = male), autism traits, intellectual ability, age of ASD diagnosis (in years), parental educational level and the presence of co-occurring psychiatric conditions.

***Autism traits*** were measured with the Dutch Autism Quotient-Short (Hoekstra et al., 2011). Informants report how well each of 28 statements describes the social behaviours, interests and preferences of the person with autism (e.g. ‘I enjoy meeting new people’). Responses are rated on a 4-point Likert-type scale ranging from 1 (definitely agree) to 4 (definitely disagree). Total AQ-Short score varies from 28 to 112, with higher scores indicating more autistic traits. The AQ-Short is highly correlated with the original 50-item AQ and has good psychometric properties (Hoekstra et al., 2011).

***Intellectual ability*** was based on self-/proxy-reported IQ at one of the seven levels, ranging from IQ below 40 (severe intellectual disability) to IQ above 130 (gifted). First, informants were asked whether they (or the person with autism if a legal representative filled in the survey) had ever taken an IQ test or whether their IQ had ever been determined. If so, informants were asked to tick one of the seven IQ levels matching the IQ test score. If the adult with autism had never taken an IQ test, did not know the IQ test score or had a very uneven IQ profile, then informants were asked to estimate the intellectual ability by ticking the appropriate IQ level. In the current study, 65% of IQ reports were based on a prior IQ test. The proportion of test-based IQs ranged from 52% (in the group of participants with a reported IQ of 85–115) to 95% (in the group with a reported IQ of 55–70). Werkman et al. (2020) found a high correlation (*r* = −0.71) between proxy-reported IQ and adaptive functioning, providing initial support for the validity of proxy-reported IQ.

***Parental education*** was based on the highest level of education successfully completed by the parent. Following the categorization system of Statistics Netherlands (CBS), this was rated as low (e.g. pre-vocational secondary school), middle (e.g. secondary vocational education) and high (e.g. university). Both maternal and paternal educational levels were assessed.

***Presence of co-occurring psychiatric conditions*** was measured by asking if the person with autism had any co-occurring psychiatric diagnosis. Response categories are: ‘Yes’, ‘No’ or ‘Don’t know/Unknown’. ‘Yes’ responses were followed up with a question about the type of diagnosis/diagnoses. A code of ‘1’ was assigned if the autistic participant had any co-occurring psychiatric condition at one or more waves; other categories were coded ‘0’.

### Procedure

Participants can join the NAR freely, via a website, at any given point. Upon registration, they give written informed consent. From that point onwards, people are invited each year to complete an online survey (with the exception of 2014, when no survey was distributed). Data described in this article were collected in five waves from 2013 (i.e. T0) to 2018 (i.e. T4). This study only considers data from autistic adults aged 18+. If the adult was not able to complete the survey independently, it could be filled in by a legal representative (often a parent). The NAR has been reviewed and approved by the ethics committee of the Vrije Universiteit Amsterdam (VCWE 2020-041R1). Preregistration of this study can be found at Open Science Framework (https://osf.io/gt38c).

### Community involvement

Autistic individuals were not actively involved in the design of this particular study, but some have reflected on the dissemination of the research findings via a stakeholder panel. In general, the content and formulation of the annual NAR survey is inspired by needs and interests expressed by stakeholders from the autism community, including autistic adults, parents and the Dutch Association for Autism (NVA), an autism advocacy group. Each year the NAR invites a panel of stakeholders (autistic adults and parents of children with varying abilities) to discuss and exchange ideas on relevant research topics, methodology and dissemination of findings. The NAR also has several autistic team members.

### Data analysis

Latent growth curve models (LGMs) were used with the intercept representing the initial level (centred at the first wave; T0) of objective or subjective functioning, and the slope term representing increases or decreases from the intercept with each wave.

**Figure 1.**
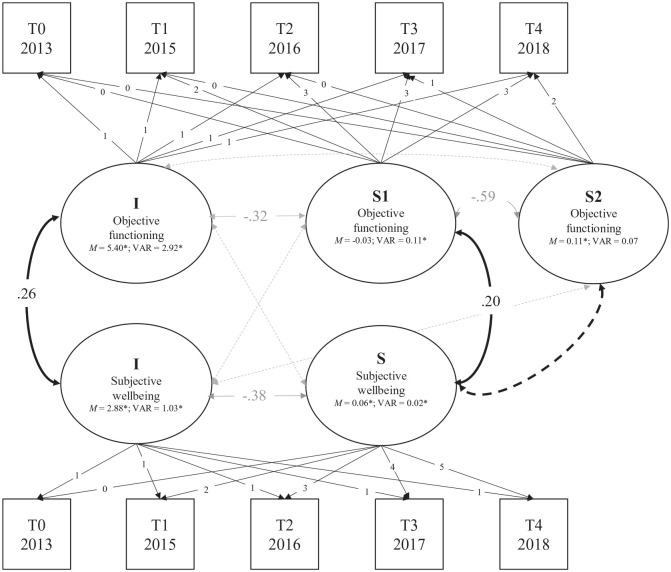
Graphical representation of the (piecewise) parallel-process LGM (measurement and structural model) between objective psychosocial functioning and subjective well-being. I = intercept, S = slope. Estimates are correlations (standardized *B*s) with *p* < 0.05, except for means and variances. Dashed lines represent non-significant associations. Black lines represent associations of interest. Grey lines are estimated in the model, but not of primary interest. *M* = latent factor mean, *VAR* = latent factor variance (unstandardized estimates). The model has approximate fit to the data, χ^2^(35) = 79.694, *p* < 0.001; CFI = 0.986; RMSEA = 0.037, 90% CI = 0.026–0.048; SRMR = 0.034.

We first fitted LGMs for the two outcomes separately to assess the number of growth parameters needed (i.e. linear or non-linear slope). Potential gender differences in the means of the intercepts and slopes of the outcomes were tested. We then investigated associated initial levels and change between objective psychosocial functioning and subjective well-being using parallel-process latent growth models (see Figure 1 for the measurement and structural model).

To investigate which factors predict initial levels and/or change over time in objective and subjective outcomes, the intercept (i.e. initial level) and slope (i.e. change) parameters of the two outcomes were regressed on the predictors and (for interactions tests) interactions between predictors and age. All predictors were added simultaneously to the model to account for potential shared variance between the predictors.

The model was fitted in Mplus version 8.0, Los Angeles, CA (Muthén & Muthén, 1998-2017). Models were estimated using the robust maximum likelihood estimator (MLR) to account for non-normality of data. Missing data were handled using full information maximum likelihood estimation (FIML). Model fit was determined using the Chi-Square Test of Model Fit, the Confirmative Fit Index (CFI, critical value ⩾ 0.95; Marsh et al., 2004) the Root Mean Square Error of Approximation (RMSEA, critical value ⩽ 0.06, Marsh et al., 2004) and the Standardized Root Mean Square Residuals (SRMR; with critical value ⩽ 0. 08; Asparouhov & Muthén, 2018).

## Results

### Descriptive Statistics

#### Outcome measures

On the *objective* psychosocial measure, averaged across the five waves, 32.6% of the adults demonstrated a good or very good outcome, 53.5% had a fair outcome, and 13.9% had a poor outcome (see Supplementary Table S1). On the *subjective* measure, an intermediate level (3) of subjective well-being was reported. At each wave, the proportion of people reporting to be (almost) always happy (7%–11%) was similar to the proportion reporting to be (almost) always unhappy (7%–9%). Pearson correlations between outcome measures at each wave and predictors are shown in Supplementary Table S2.

#### Predictor variables

In line with their clinical ASD diagnoses, the majority (93.5%) of the sample had an AQ-Short score of 65 or higher (Hoekstra et al., 2011). The mean age of ASD diagnosis was 33.8 years (see Table 2). Corresponding with their generally high educational level, over half the cohort had a high reported intellectual ability (IQ > 115). Around two-thirds (65%) of participants had (had) a co-occurring psychiatric diagnosis at one (or more) wave(s) (72% of all women vs 58% of all men). Most frequently reported diagnoses across all waves were mood disorders (women: 38%–55%; men: 30%–45%), anxiety disorders including obsessive-compulsive disorder (women: 19%–25%; men: 20%–26%), and attention deficit hyperactivity disorder (ADHD) (women: 25%–28%; men: 21%–29%).

**Table 2.** Descriptive statistics of the predictor variables.

Variable	N	M	SD	Minimum	Maximum
Age at most recent wave (in years)	917	43.5	12.4	19.0	67.9
Age of ASD diagnosis (in years)	831	33.8	14.8	2.3	63.1
AQ-Short (total)	790	82.9	11.4	46.0	110.0
	N	%			
Gender					
Female	492	53.7			
Male	425	46.3			
Intellectual ability					
IQ < 85	78	8.6			
IQ 85–115	271	29.8			
IQ > 115	560	61.6			
Co-occurring psychiatric condition(s)					
Yes	598	65.2			
No	319	34.8			
Educational level mother^1^					
Low	428	53.4			
Middle	195	24.3			
High	179	22.3			
Educational level father^1^					
Low	296	37.5			
Middle	183	23.2			
High	311	39.4			

Note. ^1^The highest level of education successfully completed by the parent was rated as low (e.g. pre-vocational secondary school), middle (e.g. secondary vocational education) or high (e.g. university).

### Unconditional LGMs of Objective Psychosocial Functioning and Subjective Well-being

We first fitted unconditional LGMs for each construct separately. Means and variances of the growth parameters for the unconditional LGMs are shown in Figure 1. Parameter estimates indicate that the development of objective psychosocial functioning was best captured by a piecewise (rather than linear) growth curve consisting of two significantly different linear slopes. The first slope showed stability in objective functioning from T0 to T2 (*B* = −0.024, SE = 0.018, *p* = 0.179), and the second slope showed an increase in objective functioning from T2 to T4 (*B* = 0.105, SE = 0.026, *p* < 0.001). The development of subjective well-being was best captured by a linear growth curve which showed that subjective well-being increased over the years (*B* = 0.055, SE = 0.009, *p* < 0.001). Furthermore, Satorra–Bentler chi-square tests showed no gender differences in mean initial levels (intercepts) or mean changes over time (slopes) for objective psychosocial functioning (Δχ^2^(3) = 3.547, *p* = 0.315) and subjective well-being (Δχ^2^(2) = 0.914, *p* = 0.633).

Next, we fitted a parallel-process unconditional LGM to test the associations between the initial levels and changes of objective psychosocial functioning and subjective well-being. There was a small positive association between initial levels of objective and subjective outcomes (*r* = 0.263; *B* = 0.455, SE = 0.086, *p* < 0.001). Change in objective psychosocial functioning from T0 to T2 (slope 1) was also (weakly) positively correlated with a change in subjective well-being (*r* = 0.200; *B* = 0.010, SE = 0.005, *p* = 0.032), but a change in objective functioning from T2 to T4 (slope 2) was not associated with a change in subjective well-being (*p* = 0.117). See Figure 1 for a graphical presentation of the results.

### Main effects of the Predictor Variables on Psychosocial Outcomes

Because chronological age and age of ASD diagnosis were highly correlated (*r* = 0.91; see Supplement Table S2), we removed ‘age of ASD diagnosis’ as a predictor. Predictor values are centred to ease interpretation.

**Figure 2.**
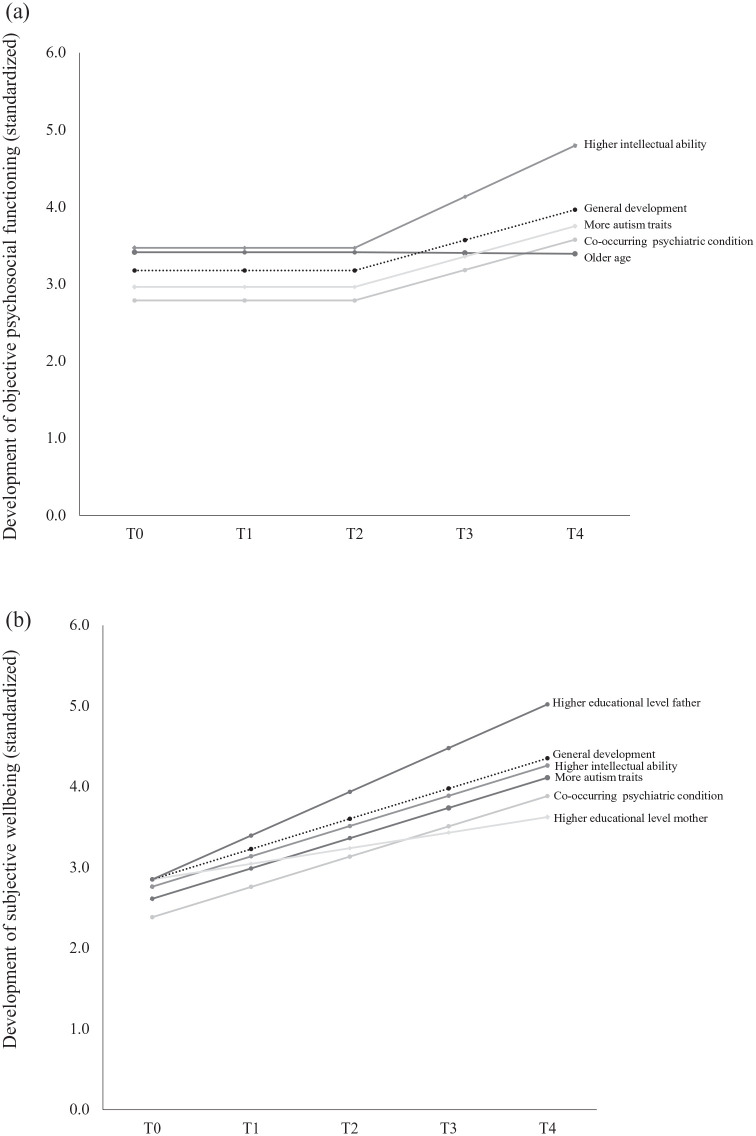
Main effects of predictors on the (standardized) level and development of objective psychosocial functioning (a) and subjective well-being (b). The dashed line represents the general developmental trend of the outcomes. Deviations from the general level of functioning at T0 represent main effects on the intercept. Deviations from the general developmental trend from T0 to T4 represent main effects on the slope. Estimates are standardized *B*s, β.

Chronological age, gender, autism traits, intellectual ability, parental educational levels and presence of co-occurring psychiatric conditions together explained 23%, 0.04% and 17% of the variance in the intercept, slope 1 and slope 2 of objective psychosocial functioning, respectively. Having a co-occurring psychiatric diagnosis and more autism traits predicted lower levels of objective psychosocial functioning at T0 (co-occurring condition: β = −0.185, *B* = −0.664, SE = 0.122, *p* < 0.001; autism traits: β = −0.213, *B* = −0.032, SE = 0.006, *p* < 0.001), while higher intellectual ability and older age predicted higher levels of objective psychosocial functioning at T0 (intellectual ability: β = 0.294, *B* = 0.435, SE = 0.052, *p* < 0.001; age: β = 0.237, *B* = 0.033, SE = 0.006, *p* < 0.001). None of the predictors predicted change in objective psychosocial functioning from T0 to T2. However, having a higher intellectual ability predicted more growth in objective psychosocial functioning from T2 to T4 compared to the general trend (β = 0.269, *B* = 0.065, SE = 0.022, *p* = 0.003), while older age predicted less growth from T2 to T4 (β = −0.404, *B* = −0.009, *SE* = 0.003, *p* < 0.001). These results are graphically represented in Figure 2(a).

For subjective well-being, chronological age, gender, autism traits, intellectual ability, parental educational levels and the presence of co-occurring psychiatric conditions together explained 13% and 5% of the variance of the intercept and slope, respectively. Again, having a co-occurring psychiatric condition and more autism traits predicted lower levels of subjective well-being at T0 (co-occurring condition: β = −0.223 *B* = −0.472, SE = 0.087, *p* < 0.001; autism traits: β = −0.240, *B* = −0.021, SE = 0.004, *p* < 0.001). In addition, higher intellectual ability also predicted lower levels of subjective well-being at T0 (β = −0.090, *B* = −0.078, SE = 0.040, *p* = 0.049). Furthermore, a higher level of maternal education predicted less growth in well-being over time, whereas a higher paternal level of education predicted more growth in well-being (educational level of mother: β = −0.182, *B* = −0.033, SE = 0.016, *p* = 0.035; educational level of father: β = 0.167, *B* = 0.029, SE = 0.014, *p* = 0.048). These results are graphically represented in Figure 2(b).

As an additional check of the robustness of our findings, we repeated the LGM with ‘age of ASD diagnosis’ as a predictor instead of ‘age’. All main results remained similar, with the exception of a now non-significant effect of intellectual ability on the intercept of subjective well-being (*p* = 0.252). Detailed coefficients (*B*, SE of *B*, 95% CI of B and standardized betas) for all predictors for both outcome variables are reported in Supplementary Tables S3 (with age as predictor) and S5 (with age of diagnosis as predictor).

### Moderation by age

**Figure 3.**
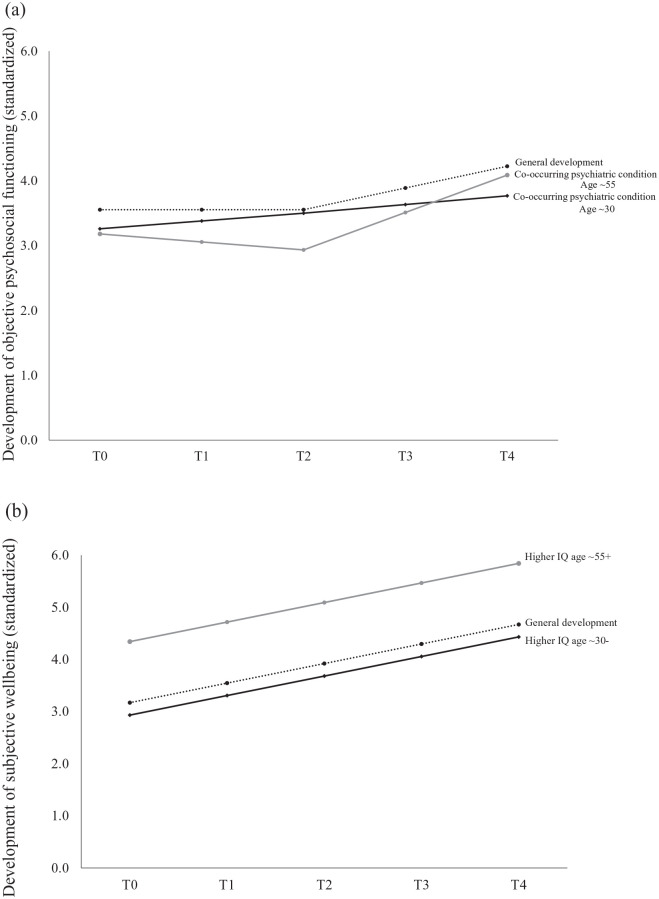
Interaction effect between having a co-occurring psychiatric disorder and age on the development of objective psychosocial functioning (a) and of having a higher intellectual ability and age on the development of subjective well-being (b) across T0–T4 for younger (black line; aged ~30 years and younger) and older (grey line; aged ~55 years and older) people diagnosed with autism (a). Deviations from the general level of functioning at T0 represent effects on the intercept. Deviations from the general developmental trend from T0 to T4 represent effects on the slope. The dashed line represents the general development of objective psychosocial functioning for the sample in total. Estimates are standardized *B*s and β.

Before the analyses, continuous variables were standardized to ease interpretation and avoid multicollinearity. Significant interaction effects of age × co-occurring psychiatry on the slopes of objective psychosocial functioning added 18% and 48% of explained variance to slope 1 and slope 2, respectively, resulting in a total of 18% and 65% of explained variance. For participants of ~30 years and younger (*M*_age_ – 1 SD), a co-occurring psychiatric condition was associated with an increase in objective psychosocial functioning from T0 to T2 (β = 0.120, SE = 0.060, *p* = 0.045), and then a decrease from T2 to T4 (β = −0.201, SE = 0.092, *p* = 0.029). In contrast, participants aged 55 and older (*M*_age_ + 1 *SD*) with a co-occurring psychiatric condition initially showed a decrease in psychosocial functioning from T0 to T2 (β *B* = −0.123, SE = 0.046, *p* = 0.007) followed by an increase from T2 to T4 (β = 0.242, SE = 0.062, *p* < 0.001), see also Figure 3(a).

An age × intellectual ability interaction effect on the initial level (intercept) of subjective well-being added 2% of explained variance, resulting in a total of 15% explained variance. For participants of ~30 years and younger (*M*_age_ – 1 SD), a higher intellectual ability was associated with a lower level of subjective well-being at T0 (β = −0.149, SE = 0.057, *p* = 0.008), while for participants aged ~55 years and older (*M*_age_ + 1 SD), intellectual ability was not associated with subjective well-being at T0 (*p* = 0.155), see also Figure 3(b).

We repeated the LGM with ‘age of ASD diagnosis’ instead of ‘age’ as a predictor and moderator. Most results remained similar, but two additional interaction effects were found on the intercept of objective psychosocial functioning and one interaction effect on the slope of subjective well-being. For participants diagnosed at an older age (*M*_age of diagnosis_ + 1 SD), a higher level of maternal education was associated with a higher initial level of objective psychosocial functioning (β = 0.260, SE = 0.115, *p* = 0.024), but this association was not found in adults diagnosed at a younger age (*M*_age of diagnosis_ – 1 SD; *p* = 0.382). Furthermore, for participants diagnosed at a younger age, higher intellectual ability was associated with a higher initial level objective psychosocial functioning (*B* = 0.370, SE = 0.071, *p* < 0.001), but this positive association was not found in participants diagnosed at an older age (*p* = 0.242). Finally, men who were diagnosed with ASD at a later age showed a greater increase in their subjective well-being over time compared to men diagnosed at an earlier age (*B* = 0.064, SE = 0.027, *p* = 0.018), whereas the effect of age of diagnosis was not significant for women (*p* = 0.095). For estimates of all interaction effects, see Supplementary Tables S4 and S6.

## Discussion

In this 6-year longitudinal study, incorporating five waves of measurement, we aimed to identify patterns and predictors of development in objective psychosocial functioning and subjective well-being in a large group of autistic men and women. In general, participants were rated as having a fair to good level of objective psychosocial functioning. As hypothesized, we found a modest increase in objective psychosocial outcome and subjective well-being over time, although improvement in objective psychosocial functioning only occurred in the second half of the study. Initial level of and change in objective outcome also correlated positively with level of and change in subjective well-being. Predictors of a good objective outcome, explaining 23% of variance, were higher intellectual ability, absence of co-occurring psychiatric conditions, fewer autism traits and older age. Predictors of higher subjective well-being, explaining 13% of variance, were lower intellectual ability, absence of co-occurring psychiatric conditions and fewer autism traits. Men and women did not differ in initial levels of or overall change in objective and subjective outcomes over time.

In contrast to previous studies showing a relatively poor outcome for autistic adults with mixed intellectual abilities (Mason et al., 2020; Steinhausen et al., 2016), the majority of participants in the current study showed a fair to good level of psychosocial functioning. When averaged across the five measurements, 86% showed a fair to (very) good overall outcome compared to only 14% with a (very) poor outcome. Clearly, these results should be regarded in the light of the sample’s comparatively high estimated intellectual ability (evidenced also by their high educational level) as well as the late timing of their ASD diagnosis (*M* = 33 years). Previously, Gotham et al. (2015) also demonstrated better objective psychosocial outcomes for self-reporting adults with a late ASD diagnosis. A diagnosis in adulthood suggests that many individuals in the present study functioned moderately well through childhood and adolescence, leaving them undetected in their early years (Lai & Baron-Cohen, 2015). Cultural and environmental factors may also play a role. In the Netherlands, the Social Support Act (WMO 2015) promotes independent living and active participation in society by individuals with disabilities and/or psychiatric problems. Local authorities have a duty to allocate care and support to those in need. Improved awareness, support and inclusion may have helped to enhance social participation among this cohort.

Participants generally reported an intermediate level of subjective well-being (‘equally happy and unhappy’). This indicates that the adults with autism reported lower levels of subjective well-being, on average, than the general Dutch population, among whom over 80% indicate that they are happy (van Beuningen & Moonen, 2019). This is also in line with lower parent-reported subjective well-being ratings for autistic children compared to those for typically developing children (Begeer et al., 2017). As hypothesized, and consistent with findings from several other studies (Lawson et al., 2020; Mason et al., 2018; Moss et al., 2017), levels of subjective and objective functioning were positively, albeit modestly correlated. Thus, adults who had obtained normative goals such as paid (part-time) employment generally also reported higher subjective well-being. Furthermore, during the first half of the study, *change* in subjective well-being was positively correlated with *change* in objective psychosocial functioning. Societal success may promote subjective well-being and/or higher subjective well-being may increase the chances of societal success. In the general population, achieving adult-appropriate goals not only contributes to higher well-being (Schulenberg et al., 2004), but happy people also have higher chances of success in social relations, work and income (De Neve & Oswald, 2012; Lyubomirsky et al., 2005; Walsh et al., 2018). This bi-directional relationship between objective and subjective outcomes may also apply to adults with autism.

At the start of the study, objective outcomes were better for older than younger adults. The older adults may have had more time to reach certain milestones such as independent living. In contrast, in an earlier longitudinal study, individuals in middle adulthood (n = 44) showed the same or sometimes poorer outcomes compared to 20 years earlier (Howlin et al., 2013). In that study, however, all participants had been diagnosed in (early) childhood and average intellectual ability was lower than in our sample. Also, we found that older adults were less likely to show improvements in objective functioning over time, possibly because they already had a higher starting point. Improvements in subjective well-being were independent of age. Studies in the general population have reported a small increase in happiness during middle adulthood (Baird et al., 2010; Sheldon & Kasser, 2001). We found some support for a happy aging effect in autistic adults across a period of 6 years, but this effect was not stronger for middle-aged compared to young adults.

As expected (e.g. Howlin et al., 2013), adults with a higher intellectual ability showed higher initial levels and greater improvement in objective psychosocial functioning over time. However, this association was not found in late-diagnosed participants. Although they may do better during childhood and teenage years, late-diagnosed adults possibly lack adequate interventions and support and may therefore receive fewer opportunities to reach their full potential. In addition, among younger (18–30 years) but not in the oldest (55–65 years) adults, intellectual ability correlated negatively with subjective well-being. Since early adulthood is commonly characterized by many changes and challenges, such as finding a suitable job, house or spouse, differences between typically and atypically developing young adults may be more evident at this phase of life and autistic adults of higher intellectual ability may be more aware of this, possibly resulting in poorer subjective well-being.

In keeping with previous research (Deserno et al., 2018; Lin & Huang, 2019; Zimmerman et al., 2018), a co-occurring psychiatric diagnosis and more autism traits predicted lower initial levels of subjective well-being and objective psychosocial functioning. Older adults (55–65 years; on average diagnosed at 51 years) with a co-occurring psychiatric disorder showed a decrease followed by an increase in objective functioning. The initial dip could stem from mental health problems for which many late-diagnosed adults seek help (Geurts & Jansen, 2011; Jones et al., 2014). Post-diagnosis guidance, support and self-understanding (Huang et al., 2020; Leedham et al., 2020) may have helped them deal with everyday life challenges more effectively, explaining the subsequent improvement. Compared with the general developmental trend in the entire sample, younger autistic adults with a co-occurring psychiatric condition showed more improvement in their objective functioning during the first half of the study, but this then plateaued during the second half. As yet, these various developmental effects require further exploration and replication.

We did not find any gender differences in the initial level or change in objective psychosocial functioning, in line with earlier studies on employment (Sung et al., 2015; Wei et al., 2015), but in contrast to studies suggesting better social functioning in autistic women (Hull et al., 2019; Lai et al., 2017). Previous findings regarding gender differences in subjective well-being have been mixed, with some studies reporting lower quality of life ratings for women (Graham Holmes et al., 2020; Mason et al., 2018), men (Lawson et al., 2020) or similar ratings (Lin & Huang, 2019; Renty & Roeyers, 2006). After statistically controlling for factors such as intellectual ability and co-occurring psychiatric conditions, our findings suggest that autistic men and women seem quite similar in their level of subjective well-being. Furthermore, later diagnosed autistic men showed a greater increase in their subjective well-being over time compared with earlier diagnosed men. This timing effect of diagnosis was not present in women, and needs to be examined further.

Finally, earlier studies suggested that a higher socio-economic family background could increase the success of autistic adults in education and career (Taylor et al., 2015; Wei et al., 2015). In the present sample, adults with highly educated fathers were more likely to show an increase in subjective well-being over time, but not in objective functioning. In contrast, adults with highly educated mothers showed less growth in subjective well-being. A methodological explanation for this unexpected finding may be that adults with highly educated mothers had less room for improvement as they tended to report a higher initial level of subjective well-being. However, further research is needed on the potential role of parental education in autistic adults’ well-being.

The strengths of this study are its longitudinal design and large sample size including many women with autism. However, our overall conclusions cannot be readily generalized to autistic individuals with an intellectual disability and/or an early childhood ASD diagnosis. Also, it remains unclear if the conclusions apply to non-binary individuals, who were not included in this study. Furthermore, missing data analyses indicated that participants with missing data at any of the five waves had somewhat lower levels of subjective well-being at the start of the study compared to those with complete data across all five waves, indicating possible selective attrition. Another limitation is the reliance on self- or proxy-report (parental report), especially with regard to IQ, mental health and autistic symptoms. Future research should ideally include clinical diagnostic assessments and standardized measures of IQ. Also, self-reporting adults with autism may have a different perspective of their subjective well-being than parents (Hong et al., 2016). Future studies should therefore consider the role of the informant. Finally, in collaboration with autistic researchers and consultants, future studies should consider how to ensure that assessments of change in objective and subjective functioning best reflect issues that are of most relevance for the autistic community.

Overall, in comparison with previous longitudinal and cross-sectional research, our findings provide a more positive outlook for autistic men and women with average to high intellectual abilities in countries with relatively high levels of support by showing a fair to good level of objective psychosocial functioning, a small increase in objective functioning and subjective well-being over 6 years, and better objective outcomes for older than young autistic adults.

## Supplemental Material

sj-docx-1-aut-10.1177_13623613211027673 – Supplemental material for Objective and subjective psychosocial outcomes in adults with autism spectrum disorder: A 6-year longitudinal study Click here for additional data file. Supplemental material, sj-docx-1-aut-10.1177_13623613211027673 for Objective and subjective psychosocial outcomes in adults with autism spectrum disorder: A 6-year longitudinal study by Anke M Scheeren, J Marieke Buil, Patricia Howlin, Meike Bartels and Sander Begeer in Autism

## Notes

1. In this article, the terms autism and ASD are used interchangeably; we also use a combination of ‘person first’ language (e.g. ‘people with autism’) and ‘identity first’ language (e.g. ‘autistic people’) to reflect the different views on terminology among those in the autism community, parents and practitioners (cf. Kenny et al., 2015).
