# Supplementary material for: Objective and subjective psychosocial outcomes in adults with autism spectrum disorder: A 6-year longitudinal study
Source: Autism. 2021 Jun 25;26(1):243–55. doi: 10.1177/13623613211027673 (PMC8750151; doi:10.1177/13623613211027673)
Supplement: sj-docx-1-aut-10.1177_13623613211027673 – Supplemental material for Objective and subjective psychosocial outcomes in adults with autism spectrum disorder: A 6-year longitudinal study [file sj-docx-1-aut-10.1177_13623613211027673.docx]

**Supplementary Material**

**Table S1.**

*Descriptive Statistics of Objective Psychosocial Functioning and Subjective Wellbeing at Each Wave*

| Variable Wave | *N* | *M* | *SD* | % very good outcome^*^ | % good outcome | % fair outcome | % poor outcome | % very poor outcome^*^ |
| --- | --- | --- | --- | --- | --- | --- | --- | --- |
| Objective psychosocial functioning (0-8) |  |  |  |  |  |  |  |  |
| 2013 | 461 | 5.38 | 1.72 | 10.4 | 17.4 | 58.3 | 13.9 | 0.0 |
| 2015 | 423 | 5.84 | 1.72 | 16.3 | 24.1 | 47.3 | 11.8 | 0.5 |
| 2016 | 566 | 5.34 | 1.70 | 9.2 | 19.8 | 56.2 | 14.6 | 0.2 |
| 2017 | 647 | 5.41 | 1.75 | 9.3 | 21.9 | 52.7 | 16.1 | 0.0 |
| 2018 | 583 | 5.55 | 1.75 | 11.1 | 23.7 | 52.7 | 12.3 | 0.2 |
| Subjective wellbeing (1-5) |  |  |  |  |  |  |  |  |
| 2013 | 489 | 2.93 | 1.11 | 7.2 |  |  |  | 8.8 |
| 2015 | 540 | 3.09 | 1.13 | 8.0 |  |  |  | 8.5 |
| 2016 | 616 | 3.10 | 1.14 | 9.3 |  |  |  | 8.0 |
| 2017 | 715 | 3.11 | 1.11 | 8.3 |  |  |  | 7.4 |
| 2018 | 648 | 3.13 | 1.16 | 11.0 |  |  |  | 8.2 |

*Note.* A very good outcome indicates the highest possible score on the scale; a very poor outcome indicates the lowest possible score.

| **Table S2**  *Correlations Between Objective and Subjective Psychosocial Outcomes and Predictor Variables* | | | | | | | | | | | | | |
| --- | --- | --- | --- | --- | --- | --- | --- | --- | --- | --- | --- | --- | --- |
|  | 1 | 2 | 3 | 4 | 5 | 6 | 7 | 8 | 9 | 10 | 11 | 12 | 13 |
| 1. Objective psychosocial functioning (2013) |  |  |  |  |  |  |  |  |  |  |  |  |  |
| 1. Objective psychosocial functioning (2015) | **.777** |  |  |  |  |  |  |  |  |  |  |  |  |
| 1. Objective psychosocial functioning (2016) | **.789** | **.782** |  |  |  |  |  |  |  |  |  |  |  |
| 1. Objective psychosocial functioning (2017) | **.818** | **.817** | **.859** |  |  |  |  |  |  |  |  |  |  |
| 1. Objective psychosocial functioning (2018) | **.795** | **.748** | **.742** | **.777** |  |  |  |  |  |  |  |  |  |
| 1. Subjective wellbeing (2013) | **.220** | **.244** | .114 | .144 | .108 |  |  |  |  |  |  |  |  |
| 1. Subjective wellbeing (2015) | **.240** | **.335** | **.183** | .148 | .178 | **.732** |  |  |  |  |  |  |  |
| 1. Subjective wellbeing (2016) | **.202** | **.281** | **.241** | **.179** | .142 | **.598** | **.735** |  |  |  |  |  |  |
| 1. Subjective wellbeing (2017) | **.208** | **.295** | **.225** | **.205** | **.178** | **.558** | **.697** | **.729** |  |  |  |  |  |
| 1. Subjective wellbeing (2018) | .131 | **.281** | **.180** | **.171** | **.166** | **.580** | **.632** | **.647** | **.730** |  |  |  |  |
| 11. Autism traits | **-.205** | **-.244** | **-.155** | **-.169** | **-.188** | **-.269** | **-.297** | **-.198** | **-.204** | **-.187** |  |  |  |
| 1. Intellectual ability | **.368** | .157 | **.330** | **.394** | **.407** | -.108 | -.086 | -.047 | -.028 | -.037 | .059 |  |  |
| 1. Age | **.267** | **.189** | **.262** | **.267** | **.186** | -.064 | -.095 | -.084 | -.057 | -.088 | **.156** | **.294** |  |
| 1. Age of diagnosis | **.328** | **.220** | **.293** | **.334** | **.253** | -.127 | -.104 | -.111 | -.116 | -.133 | **.188** | **.358** | **.907** |

***Note.*** Estimates in bold are significant at two-sided *p* ≤ .000.

**Table S3**.

*Main Effects of Predictors on the Growth Parameters of Objective Psychosocial Functioning and Subjective Wellbeing*

|  |  |  | *B* | *SE* | *p* | 95% CI of *B* | | β |
| --- | --- | --- | --- | --- | --- | --- | --- | --- |
|  | |  |  |  |  | UL | LL |  |
|  |  | Intercept Objective Psychosocial Functioning | | | | | | |
|  | Gender |  | -.152 | .121 | .208 | -.389 | .085 | -.089 |
|  | Comorbid diagnosis (y/n) |  | **-.664** | **.122** | **<.001** | **-.903** | **-.424** | **-.388** |
|  | Educational level mother |  | .019 | .096 | .842 | -.170 | .208 | .009 |
|  | Educational level father |  | .035 | .089 | .692 | -.140 | .210 | .018 |
|  | Intellectual ability |  | **.435** | **.052** | **<.001** | **.333** | **.536** | **.294** |
|  | Age |  | **.033** | **.006** | **<.001** | **.021** | **.044** | **.237** |
|  | Autism traits (*n* symptoms) |  | **-.032** | **.006** | **<.001** | **-.043** | **-.021** | **-.213** |
|  | Total explained variance |  | .233 | | | | | |
|  |  | Slope 1 Objective psychosocial Functioning | | | | | | |
|  | Gender |  | -.012 | .036 | .750 | -.083 | .059 | -.034 |
|  | Comorbid diagnosis (y/n) |  | -.027 | .035 | .448 | -.095 | .042 | -.079 |
|  | Educational level mother |  | -.009 | .032 | .765 | -.071 | .052 | -.023 |
|  | Educational level father |  | .002 | .028 | .937 | -.053 | .057 | .006 |
|  | Intellectual ability |  | -.014 | .015 | .358 | -.042 | .015 | -.047 |
|  | Age |  | .000 | .002 | .899 | -.003 | .004 | .009 |
|  | Autism traits (*n* symptoms) |  | .000 | .002 | .951 | -.003 | .003 | -.003 |
|  | Total explained variance |  | .004 | | | | | |
|  |  | Slope 2 Objective Psychosocial Functioning | | | | | | |
|  | Gender |  | -.036 | .051 | .482 | -.136 | .064 | -.127 |
|  | Comorbid diagnosis (y/n) |  | .041 | .052 | .432 | -.061 | .142 | .144 |
|  | Educational level mother |  | -.044 | .038 | .249 | -.119 | .031 | -.127 |
|  | Educational level father |  | -.008 | .035 | .817 | -.077 | .061 | -.025 |
|  | Intellectual ability |  | **.065** | **.022** | **.003** | **.023** | **.108** | **.269** |
|  | Age |  | **-.009** | **.003** | **<.001** | **-.014** | **-.004** | **-.404** |
|  | Autism traits (*n* symptoms) |  | .001 | .002 | .663 | -.003 | .005 | .040 |
|  | Total explained variance |  | .168 | | | | | |
|  |  | Intercept Subjective Wellbeing | | | | | | |
|  | Gender |  | -.021 | .085 | .803 | -.188 | .146 | -.021 |
|  | Comorbid diagnosis (y/n) |  | **-.472** | **.087** | **<.001** | **-.644** | **-.301** | **-.468** |
|  | Educational level mother |  | .126 | .067 | .061 | -.006 | .258 | .102 |
|  | Educational level father |  | -.040 | .063 | .524 | -.164 | .084 | -.035 |
|  | Intellectual ability |  | **-.078** | **.040** | **.049** | **-.156** | **-0.00** | **-.090** |
|  | Age |  | .000 | .004 | .974 | -.007 | .008 | .001 |
|  | Autism traits (*n* symptoms) |  | **-.021** | **.004** | **<.001** | **-.029** | **-.013** | **-.240** |
|  | Total explained variance |  | .132 | | | | | |
|  |  |  | Slope Subjective Wellbeing | | | | | |
|  | Gender |  | .009 | .018 | .641 | -.028 | .045 | .057 |
|  | Comorbid diagnosis (y/n) |  | .002 | .020 | .921 | -.037 | .041 | .013 |
|  | Educational level mother |  | **-.033** | **.016** | **.035** | **-.065** | **-.002** | **-.182** |
|  | Educational level father |  | **.029** | **.014** | **.048** | **.000** | **.057** | **.167** |
|  | Intellectual ability |  | .013 | .008 | .121 | -.003 | .029 | .098 |
|  | Age |  | -.001 | .001 | .360 | -.002 | .001 | -.060 |
|  | Autism traits (*n* symptoms) |  | .001 | .001 | .462 | -.001 | .002 | .050 |
|  | Total explained variance |  | .047 | | | | | |

*Note.* CFI = .973. RMSEA = .037. 90% confidence interval = .030 (lower limit) - .044 (upper limit); SRMR = .035. Estimates in bold are significant at two-sided *p* ≤ .05

**Table S4.**

*Interaction Effects Between the Predictors and Age on the Growth Parameters of Objective Psychosocial Functioning and Subjective Wellbeing*

|  |  |  | *B* | *SE* | *p* | 95% CI of *B* | | β |
| --- | --- | --- | --- | --- | --- | --- | --- | --- |
|  | |  |  |  |  | UL | LL |  |
|  |  | Intercept Objective Psychosocial Functioning | | | | | | |
|  |  | Main effect | | | | | | |
|  | Gender |  | -.117 | .126 | .352 | -.363 | .129 | -.070 |
|  | Comorbid diagnosis (n/y) |  | **-.668** | **.127** | **<.001** | **-.916** | **-.420** | **-.398** |
|  | Educational level mother |  | .046 | .078 | .559 | -.107 | .198 | .027 |
|  | Educational level father |  | .021 | .081 | .800 | -.138 | .180 | .012 |
|  | Intellectual ability |  | **.437** | **.069** | **<.001** | **.302** | **.572** | **.260** |
|  | Age |  | **.261** | **.132** | **.048** | **.002** | **.519** | **.155** |
|  | Autism traits (*n* symptoms) |  | **-.360** | **.069** | **<.001** | **-.496** | **-.225** | **-.215** |
|  |  |  | Interaction effect | | | | | |
|  | Gender * age |  | .119 | .128 | .351 | -.132 | .370 | .051 |
|  | Comorbid diagnosis * age |  | .109 | .126 | .387 | -.137 | .355 | .052 |
|  | Educational level mother * age |  | .148 | .083 | .076 | -.015 | .311 | .085 |
|  | Educational level father * age |  | .096 | .084 | .255 | -.069 | .261 | .054 |
|  | Intellectual ability * age |  | -.061 | .056 | .277 | -.172 | .049 | -.042 |
|  | Autism traits * age |  | .004 | .064 | .952 | -.122 | .130 | .002 |
|  | Total explained variance |  | .218 | | | | | |
|  |  | Slope 1 Objective Psychosocial Functioning | | | | | | |
|  |  | Main effects | | | | | | |
|  | Gender |  | .007 | .037 | .845 | -.066 | .080 | .020 |
|  | Comorbid diagnosis (y/n) |  | -.002 | .036 | .965 | -.072 | .069 | -.004 |
|  | Educational level mother |  | -.009 | .025 | .733 | -.058 | .041 | -.024 |
|  | Educational level father |  | -.002 | .025 | .947 | -.051 | .048 | -.005 |
|  | Intellectual ability |  | -.016 | .018 | .384 | -.050 | .019 | -.043 |
|  | Age |  | **.100** | **.040** | **.013** | **.021** | **.179** | **.274** |
|  | Autism traits (*n* symptoms) |  | -.006 | .020 | .779 | -.046 | .034 | -.016 |
|  |  |  | Interaction effects | | | | | |
|  | Gender * age |  | -.039 | .041 | .339 | -.119 | .041 | -.076 |
|  | Comorbid diagnosis * age |  | **-.122** | **.039** | **.002** | **-.199** | **-.045** | **-.267** |
|  | Educational level mother * age |  | -.018 | .028 | .533 | -.074 | .038 | -.047 |
|  | Educational level father * age |  | .020 | .027 | .467 | -.034 | .074 | .052 |
|  | Intellectual ability * age |  | -.025 | .017 | .143 | -.058 | .008 | -.079 |
|  | Autism traits * age |  | .019 | .021 | .369 | -.022 | .059 | .052 |
|  | Total explained variance |  | .179 | | | | | |
|  |  | Slope 2 Objective Psychosocial Functioning | | | | | | |
|  |  | Main effects | | | | | | |
|  | Gender |  | -.008 | .052 | .872 | -.111 | .094 | -.022 |
|  | Comorbid diagnosis (y/n) |  | .021 | .053 | .699 | -.084 | .125 | .053 |
|  | Educational level mother |  | -.029 | .031 | .341 | -.090 | .031 | -.076 |
|  | Educational level father |  | .003 | .031 | .921 | -.058 | .065 | .008 |
|  | Intellectual ability |  | **.048** | **.024** | **.046** | **.001** | **.059** | **.124** |
|  | Age |  | **-.270** | **.058** | **<.001** | **-.385** | **-.156** | **-.700** |
|  | Autism traits (*n* symptoms) |  | .020 | .027 | .454 | -.032 | .073 | .052 |
|  |  |  | Interaction effects | | | | | |
|  | Gender * age |  | .013 | .055 | .808 | -.095 | .122 | .025 |
|  | Comorbid diagnosis * age |  | **.222** | **.057** | **<.001** | **.109** | **.334** | **.458** |
|  | Educational level mother * age |  | .049 | .033 | .140 | -.016 | .113 | .122 |
|  | Educational level father * age |  | -.036 | .035 | .300 | -.103 | .032 | -.088 |
|  | Intellectual ability * age |  | -.028 | .024 | .233 | -.075 | .018 | -.085 |
|  | Autism traits * age |  | -.029 | .026 | .273 | -.081 | .023 | -.076 |
|  | Total explained variance |  | .647 | | | | | |
|  |  | Intercept Subjective Wellbeing | | | | | | |
|  |  | Main effects | | | | | | |
|  | Gender |  | -.065 | .087 | .455 | -.235 | .105 | -.064 |
|  | Comorbid diagnosis (y/n) |  | **-.491** | **.088** | **<.001** | **-.663** | **-.319** | **-.487** |
|  | Educational level mother |  | .098 | .055 | .074 | -.009 | .205 | .097 |
|  | Educational level father |  | -.043 | .056 | .444 | -.152 | .067 | -.042 |
|  | Intellectual ability |  | -.025 | .047 | .599 | -.117 | .067 | -.024 |
|  | Age |  | .058 | .088 | .511 | -.114 | .229 | .057 |
|  | Autism traits (*n* symptoms) |  | **-.244** | **.048** | **<.001** | **-.339** | **-.150** | **-.242** |
|  |  | Interaction effects | | | | | | |
|  | Gender * age |  | -.114 | .087 | .188 | -.285 | .056 | -.081 |
|  | Comorbid diagnosis * age |  | .018 | .088 | .839 | -.155 | .191 | .014 |
|  | Educational level mother * age |  | .000 | .055 | .995 | -.107 | .107 | .000 |
|  | Educational level father * age |  | -.001 | .059 | .991 | -.117 | .116 | -.001 |
|  | Intellectual ability * age |  | **.124** | **.043** | **.004** | **.040** | **.209** | **.143** |
|  | Autism traits * age |  | .016 | .045 | .721 | -.072 | .105 | .016 |
|  | Total explained variance |  | .150 | | | | | |
|  |  |  | Slope Subjective Wellbeing | | | | | |
|  |  |  | Main effects | | | | | |
|  | Gender |  | .011 | .019 | .559 | -.026 | .047 | .072 |
|  | Comorbid diagnosis (y/n) |  | .005 | .020 | .811 | -.035 | .045 | .032 |
|  | Educational level mother |  | **-.027** | **.013** | **.030** | **-.052** | **-.003** | **-.179** |
|  | Educational level father |  | .025 | .013 | .056 | -.001 | .050 | .163 |
|  | Intellectual ability |  | .007 | .009 | .469 | -.012 | .025 | .045 |
|  | Age |  | -.021 | .020 | .294 | -.060 | .018 | -.137 |
|  | Autism traits (*n* symptoms) |  | .006 | .011 | .579 | -.015 | .028 | .040 |
|  |  |  | Interaction effects | | | | | |
|  | Gender * age |  | .031 | .019 | .099 | -.006 | .068 | .147 |
|  | Comorbid diagnosis * age |  | -.007 | .020 | .713 | -.046 | .032 | -.039 |
|  | Educational level mother * age |  | -.009 | .012 | .475 | -.032 | .015 | -.055 |
|  | Educational level father * age |  | .015 | .013 | .245 | -.010 | .041 | .095 |
|  | Intellectual ability * age |  | -.013 | .008 | .123 | -.029 | .003 | -.099 |
|  | Autism traits * age |  | .005 | .010 | .594 | -.014 | .025 | .035 |
|  | Total explained variance |  | .085 | | | | | |

*Note.* Estimates in bold are significant at two-sided *p* ≤ .05

**Table S5.**

*Main Effects of Predictors on the Growth Parameters of Objective Psychosocial Functioning and Subjective Wellbeing* *(with Age of Diagnosis)*

|  |  |  | *B* | *SE* | *p* | 95% CI of *B* | | β |
| --- | --- | --- | --- | --- | --- | --- | --- | --- |
|  | |  |  |  |  | UL | LL |  |
|  |  | Intercept Objective Psychosocial Functioning | | | | | | |
|  | Gender |  | -.078 | .118 | .510 | -.310 | .154 | -.046 |
|  | Comorbid diagnosis (y/n) |  | **-.651** | **.122** | **<.001** | **-.890** | **-.413** | **-.383** |
|  | Educational level mother |  | .070 | .097 | .474 | -.121 | .260 | .033 |
|  | Educational level father |  | .035 | .087 | .690 | -.135 | .204 | .018 |
|  | Intellectual ability |  | **.371** | **.055** | **<.001** | **.264** | **.478** | **.252** |
|  | Age of diagnosis |  | **.033** | **.005** | **<.001** | **.023** | **.043** | **.294** |
|  | Autism traits (*n* symptoms) |  | **-.032** | **.006** | **<.001** | **-.043** | **-.021** | **-.214** |
|  | Total explained variance |  | .244 | | | | | |
|  |  | Slope 1 Objective Psychosocial Functioning | | | | | | |
|  | Gender |  | -.010 | .035 | .770 | -.079 | .059 | -.031 |
|  | Comorbid diagnosis (y/n) |  | -.025 | .035 | .472 | -.095 | .044 | -.075 |
|  | Educational level mother |  | -.004 | .032 | .889 | -.067 | .058 | -.011 |
|  | Educational level father |  | .001 | .028 | .965 | -.053 | .056 | .003 |
|  | Intellectual ability |  | -.014 | .016 | .381 | -.046 | .018 | -.049 |
|  | Age of diagnosis |  | .000 | .002 | .760 | -.003 | .004 | .022 |
|  | Autism traits (*n* symptoms) |  | .000 | .002 | .857 | -.004 | .003 | -.010 |
|  | Total explained variance |  | .004 | | | | | |
|  |  | Slope 2 Objective Psychosocial Functioning | | | | | | |
|  | Gender |  | -.070 | .050 | .163 | -.168 | .028 | -.249 |
|  | Comorbid diagnosis (y/n) |  | .037 | .053 | .484 | -.066 | .140 | .131 |
|  | Educational level mother |  | -.044 | .039 | .254 | -.120 | .032 | -.128 |
|  | Educational level father |  | .007 | .035 | .848 | -.061 | .075 | -.021 |
|  | Intellectual ability |  | **.065** | **.023** | **.005** | **.019** | **.111** | **.269** |
|  | Age of diagnosis |  | **-.005** | **.002** | **.025** | **-.009** | **-.001** | **-.267** |
|  | Autism traits (*n* symptoms) |  | .001 | .002 | .767 | -.004 | .005 | .027 |
|  | Total explained variance |  | .107 | | | | | |
|  |  | Intercept Subjective Wellbeing | | | | | | |
|  | Gender |  | -.010 | .084 | .907 | -.174 | .154 | -.010 |
|  | Comorbid diagnosis (y/n) |  | **-.480** | **.087** | **<.001** | **-.650** | **-.309** | **-.475** |
|  | Educational level mother |  | .100 | .069 | .150 | -.036 | .236 | .080 |
|  | Educational level father |  | -.054 | .063 | .391 | -.177 | .069 | -.047 |
|  | Intellectual ability |  | *-.047* | *.041* | *.252* | *-.128* | *.034* | *-.054* |
|  | Age of diagnosis |  | -.006 | .003 | .084 | -.013 | .001 | -.090 |
|  | Autism traits (*n* symptoms) |  | **-.020** | **.004** | **<.001** | **-.028** | **-.012** | **-.229** |
|  | Total explained variance |  | .140 | | | | | |
|  |  |  | Slope Subjective Wellbeing | | | | | |
|  | Gender |  | .006 | .018 | .759 | -.030 | .042 | .037 |
|  | Comorbid diagnosis (y/n) |  | .003 | .020 | .897 | -.036 | .042 | .017 |
|  | Educational level mother |  | **-.033** | **.016** | **.044** | **-.065** | **-.001** | **-.179** |
|  | Educational level father |  | **.030** | **.014** | **.035** | **.002** | **.058** | **.176** |
|  | Intellectual ability |  | .011 | .009 | .212 | -.006 | .028 | .085 |
|  | Age of diagnosis |  | .000 | .001 | .870 | -.002 | .001 | -.012 |
|  | Autism traits (*n* symptoms) |  | .001 | .001 | .488 | -.001 | .002 | .047 |
|  | Total explained variance |  | .045 | | | | | |

*Note.* CFI = .980. RMSEA = .032. 90% confidence interval = .025 (lower limit) - .040 (upper limit); SRMR = .031. Estimates in bold are significant at two-sided *p* ≤ .05. Estimates in italic indicate that results are different compared to the effects of age.

**Table S6.**

*Interaction Effects Between the Predictors and Age of Diagnosis on the Growth Parameters of Objective Psychosocial Functioning and Subjective Wellbeing*

|  |  |  | *B* | *SE* | *p* | 95% CI of *B* | | β |
| --- | --- | --- | --- | --- | --- | --- | --- | --- |
|  | |  |  |  |  | UL | LL |  |
|  |  | Intercept Objective Psychosocial Functioning | | | | | | |
|  |  | Main effect | | | | | | |
|  | Gender |  | .051 | .125 | .683 | -.194 | .296 | .031 |
|  | Comorbid diagnosis (n/y) |  | **-.567** | **.125** | **<.001** | **-.812** | **-.322** | **-.343** |
|  | Educational level mother |  | .079 | .079 | .322 | -.077 | .234 | .048 |
|  | Educational level father |  | .052 | .077 | .499 | -.098 | .202 | .031 |
|  | Intellectual ability |  | **.248** | **.075** | **.001** | **.101** | **.394** | **.150** |
|  | Age of diagnosis |  | **.385** | **.138** | **.005** | **.114** | **.656** | **.233** |
|  | Autism traits (*n* symptoms) |  | **-.349** | **.067** | **<.001** | **-.479** | **-.218** | **-.211** |
|  |  |  | Interaction effect | | | | | |
|  | Gender * age of diagnosis |  | -.012 | .132 | .926 | -.272 | .247 | -.006 |
|  | Comorbid diagnosis * age of diagnosis |  | .126 | .134 | .345 | -.136 | .389 | .062 |
|  | Educational level mother * age of diagnosis |  | ***.182*** | ***.086*** | ***.034*** | ***.014*** | ***.349*** | ***.106*** |
|  | Educational level father * age of diagnosis |  | .055 | .082 | .503 | -.106 | .217 | .032 |
|  | Intellectual ability * age of diagnosis |  | ***-.122*** | ***.052*** | ***.019*** | ***-.224*** | ***-.020*** | ***-.101*** |
|  | Autism traits * age of diagnosis |  | .019 | .067 | .779 | -.113 | .150 | .011 |
|  | Total explained variance |  | .219 | | | | | |
|  |  | Slope 1 Objective Psychosocial Functioning | | | | | | |
|  |  | Main effects | | | | | | |
|  | Gender |  | -.000 | .036 | .994 | -.071 | .070 | -.001 |
|  | Comorbid diagnosis (y/n) |  | -.034 | .035 | .331 | -.102 | .034 | -.098 |
|  | Educational level mother |  | -.004 | .026 | .863 | -.055 | .046 | -.013 |
|  | Educational level father |  | .002 | .023 | .940 | -.044 | .047 | -.005 |
|  | Intellectual ability |  | -.024 | .023 | .303 | -.068 | .021 | -.068 |
|  | Age of diagnosis |  | **.085** | **.039** | **.030** | **.008** | **.161** | **.246** |
|  | Autism traits (*n* symptoms) |  | -.001 | .019 | .943 | -.039 | .036 | -.004 |
|  |  |  | Interaction effects | | | | | |
|  | Gender * age of diagnosis |  | -.012 | .040 | .760 | -.090 | .066 | -.026 |
|  | Comorbid diagnosis * age of diagnosis |  | **-.109** | **.039** | **.005** | **-.186** | **-.032** | **-.254** |
|  | Educational level mother * age of diagnosis |  | -.020 | .029 | .483 | -.077 | .036 | -.057 |
|  | Educational level father * age of diagnosis |  | .033 | .026 | .198 | -.017 | .084 | .093 |
|  | Intellectual ability * age of diagnosis |  | -.017 | .017 | .315 | -.050 | .016 | -.067 |
|  | Autism traits * age of diagnosis |  | .017 | .021 | .431 | -.025 | .059 | .048 |
|  | Total explained variance |  | .137 | | | | | |
|  |  | Slope 2 Objective Psychosocial Functioning | | | | | | |
|  |  | Main effects | | | | | | |
|  | Gender |  | -.070 | .054 | .194 | -.176 | .036 | -.203 |
|  | Comorbid diagnosis (y/n) |  | .063 | .054 | .248 | -.044 | .169 | .182 |
|  | Educational level mother |  | -.017 | .033 | .599 | -.081 | .047 | -.049 |
|  | Educational level father |  | .003 | .031 | .933 | -.058 | .063 | .008 |
|  | Intellectual ability |  | .031 | .030 | .301 | .028 | .089 | .089 |
|  | Age of diagnosis |  | **-.213** | **.063** | **.001** | **-.336** | **-.090** | **-.616** |
|  | Autism traits (*n* symptoms) |  | .017 | .028 | .551 | -.039 | .073 | .049 |
|  |  |  | Interaction effects | | | | | |
|  | Gender * age of diagnosis |  | -.023 | .058 | .694 | -.137 | .091 | -.049 |
|  | Comorbid diagnosis * age of diagnosis |  | **.213** | **.061** | **.001** | **.092** | **.333** | **.496** |
|  | Educational level mother * age of diagnosis |  | .026 | .037 | .490 | -.047 | .099 | .072 |
|  | Educational level father * age of diagnosis |  | -.018 | .038 | .630 | -.092 | .056 | -.051 |
|  | Intellectual ability * age of diagnosis |  | -.038 | .024 | .114 | -.085 | .009 | -.149 |
|  | Autism traits * age of diagnosis |  | -.043 | .030 | .144 | -.101 | .015 | -.124 |
|  | Total explained variance |  | .540 | | | | | |
|  |  | Intercept Subjective Wellbeing | | | | | | |
|  |  | Main effects | | | | | | |
|  | Gender |  | -.040 | .088 | .648 | -.212 | .132 | -.040 |
|  | Comorbid diagnosis (y/n) |  | **-.494** | **.088** | **<.001** | **-.666** | **-.322** | **-.496** |
|  | Educational level mother |  | .069 | .057 | .229 | -.043 | .181 | .069 |
|  | Educational level father |  | -.051 | .056 | .362 | -.160 | .058 | -.051 |
|  | Intellectual ability |  | .072 | .052 | .167 | -.030 | .173 | .072 |
|  | Age of diagnosis |  | -.018 | .090 | .844 | -.194 | .159 | -.018 |
|  | Autism traits (*n* symptoms) |  | **-.230** | **.047** | **<.001** | **-.322** | **-.137** | **-.230** |
|  |  | Interaction effects | | | | | | |
|  | Gender * age of diagnosis |  | -.145 | .090 | .107 | -.321 | .031 | -.108 |
|  | Comorbid diagnosis * age of diagnosis |  | .023 | .090 | .802 | -.153 | .198 | .018 |
|  | Educational level mother * age of diagnosis |  | -.038 | .060 | .528 | -.156 | .080 | -.037 |
|  | Educational level father * age of diagnosis |  | .003 | .061 | .963 | -.117 | .122 | .003 |
|  | Intellectual ability * age of diagnosis |  | **.124** | **.039** | **.001** | **.048** | **.200** | **.170** |
|  | Autism traits * age of diagnosis |  | -.002 | .050 | .970 | -.100 | .096 | -.002 |
|  | Total explained variance |  | .150 | | | | | |
|  |  |  | Slope Subjective Wellbeing | | | | | |
|  |  |  | Main effects | | | | | |
|  | Gender |  | .009 | .019 | .619 | -.028 | .046 | .060 |
|  | Comorbid diagnosis (y/n) |  | .004 | .020 | .827 | -.035 | .044 | .028 |
|  | Educational level mother |  | **-.031** | **.013** | **.022** | **-.057** | **-.004** | **-.197** |
|  | Educational level father |  | **.039** | **.013** | **.003** | .003 | .064 | .246 |
|  | Intellectual ability |  | -.007 | .012 | .572 | -.030 | .016 | -.042 |
|  | Age of diagnosis |  | -.030 | .020 | .149 | -.070 | .011 | -.189 |
|  | Autism traits (*n* symptoms) |  | .006 | .011 | .556 | -.015 | .028 | .041 |
|  |  |  | Interaction effects | | | | | |
|  | Gender * age of diagnosis |  | ***.055*** | ***.020*** | ***.005*** | ***.017*** | ***.093*** | ***.262*** |
|  | Comorbid diagnosis * age of diagnosis |  | -.003 | .020 | .899 | -.041 | .036 | -.013 |
|  | Educational level mother * age of diagnosis |  | .005 | .014 | .695 | -.022 | .033 | .034 |
|  | Educational level father * age of diagnosis |  | .004 | .014 | .759 | -.023 | .032 | .026 |
|  | Intellectual ability * age of diagnosis |  | -.013 | .009 | .132 | -.031 | .004 | -.116 |
|  | Autism traits * age of diagnosis |  | .001 | .011 | .921 | -.021 | .023 | .007 |
|  | Total explained variance |  | .162 | | | | | |

*Note.* Estimates in bold are significant at two-sided *p* ≤ .05. Estimates in italic indicate that results are different compared to the effects of age.
